# The Effect of Prior Knowledge of Color on Behavioral Responses and Event-Related Potentials During Go/No-go Task

**DOI:** 10.3389/fnhum.2021.674964

**Published:** 2021-06-10

**Authors:** Nami Kubo, Tatsunori Watanabe, Xiaoxiao Chen, Takuya Matsumoto, Keisuke Yunoki, Takayuki Kuwabara, Hikari Kirimoto

**Affiliations:** ^1^Department of Sensorimotor Neuroscience, Graduate School of Biomedical and Health Sciences, Hiroshima University, Hiroshima, Japan; ^2^Research Fellow of Japan Society for the Promotion of Science, Tokyo, Japan

**Keywords:** N2, P3, Go/No-go task, reaction time, prior knowledge of color, event-related potential, Stroop

## Abstract

In daily life, the meaning of color plays an important role in execution and inhibition of a motor response. For example, the symbolism of traffic light can help pedestrians and drivers to control their behavior, with the color green/blue meaning go and red meaning stop. However, we don’t always stop with a red light and sometimes start a movement with it in such a situation as drivers start pressing the brake pedal when a traffic light turns red. In this regard, we investigated how the prior knowledge of traffic light signals impacts reaction times (RTs) and event-related potentials (ERPs) in a Go/No-go task. We set up Blue Go/Red No-go and Red Go/Blue No-go tasks with three different go signal (Go) probabilities (30, 50, and 70%), resulting in six different conditions. The participants were told which color to respond (Blue or Red) just before each condition session but didn’t know the Go probability. Neural responses to Go and No-go signals were recorded at Fz, Cz, and Oz (international 10–20 system). We computed RTs for Go signal and N2 and P3 amplitudes from the ERP data. We found that RT was faster when responding to blue than red light signal and also was slower with lower Go probability. Overall, N2 amplitude was larger in Red Go than Blue Go trial and in Red No-go than Blue No-go trial. Furthermore, P3 amplitude was larger in Red No-go than Blue No-go trial. Our findings of RT and N2 amplitude for Go ERPs could indicate the presence of Stroop-like interference, that is a conflict between prior knowledge about traffic light signals and the meaning of presented signal. Meanwhile, the larger N2 and P3 amplitudes in Red No-go trial as compared to Blue No-go trial may be due to years of experience in stopping an action in response to a red signal and/or attention. This study provides the better understanding of the effect of prior knowledge of color on behavioral responses and its underlying neural mechanisms.

## Introduction

Execution and inhibition of voluntary movements are often influenced by the meaning of colors in contextually relevant situations. For instance, the color green/blue means go while the color red means stop in the traffic control system, which guides our behavior during walking and driving. According to Peschke et al. (2013), color of the traffic lights influences pedestrian’s behavior more than the object shape. However, it is currently unclear how individuals make responses to a signal when the meanings of colors are opposite to those in the traffic control system (i.e., the color green/blue means stop while the color red means go). As drivers need to *start* pressing the brake pedal when a traffic light turns red to avoid traffic accidents, it is important to understand how the prior knowledge of traffic light color impacts behavioral responses and the underlying neural mechanisms.

There are several studies that examined the effect of color on reaction times (RTs). For example, Eason et al. (1967) showed comparable reaction times for blue or red lights in a simple RT task. Also, Anllo-Vento et al. (1998) investigated attention to red-and-gray and blue-and-gray checkerboards using a task in which participants pressed a button when a dimmer target of attended color was detected, and found that RTs were similar for red and blue checkboards. On the other hand, in a visual search task, stimulus discrimination time was revealed to be faster for red than blue and yellow stimuli (Pomerleau et al., 2014). In addition, Hochman et al. (2018) investigated the effect of color on RTs using a stop-signal task, in which participants were required to respond to a traffic light picture (green and red) and had to stop the initiated response when an auditory stop signal was presented in some trials. They found that RTs in trials without the stop signal were faster with green than red traffic light picture, whereas stop-signal RTs were faster with red than green traffic light picture. Collectively, these studies indicate that there is the effect of color on behavioral responses in a relatively difficult tasks, especially when the task requires the ability to stop an ongoing action; however, there is no study investigating the effect of color on the ability to inhibit a response proactively.

One of the tasks that examine proactive response inhibition is a Go/No-go task. In the task, participants are required to respond when a target signal is presented but have to refrain from responding when a non-target signal is presented. To understand the neural mechanisms underlying the behavioral responses, brain activity is often recorded by means of event-related potentials (ERPs), which are well used to investigate the processing of exogenous and endogenous events due to the high temporal resolution. The first major ERP component observed mainly at the occipital site around 50–100 ms is commonly called C1 and originates in the primary visual cortex (Clark and Hillyard, 1996). The C1 has been considered to be unaffected by attention (Clark and Hillyard, 1996; Anllo-Vento et al., 1998), but by exogenous factors such as stimulus color (Anllo-Vento et al., 1998).

Following the C1, there are two major ERP components that are associated with cognitive processes. The first one is called N2, a negative component observed around 200 ms after the stimulus presentation. The N2 has been thought to be related to response inhibition because it is typically larger for a non-target than target signal at front-central sites (Folstein and Van Petten, 2008). Meanwhile, there is an argument that the N2 reflects conflict control process rather than response inhibition (Nieuwenhuis et al., 2003; Donkers and Van Boxtel, 2004; Enriquez-Geppert et al., 2010). In any case, the main source of N2 is estimated to be at the anterior cingulate cortex (ACC) (Nieuwenhuis et al., 2003).

The second one is called P3, which occurs following the N2 around 300 ms after the stimulus presentation. Although P3 has been reported to reflect a number of different cognitive mechanisms, such as confidence (Addante, 2015), novelty processing (Knight and Scabini, 1998), metacognition (Muller et al., 2021), and decision making (Boldt et al., 2019), here we focus on ones related to Go/No-go task. Specifically, Enriquez-Geppert et al. (2010) investigated the influence of conflict and inhibition on N2 and P3 using a combined Go/No-go and stop-signal task, during which the degree of conflict was manipulated by varying probability of go signal (75 vs. 25%) while inhibition was evaluated by three signals, Go, No-go and stop. They reported the larger P3 amplitude in stop than Go trial and found a minor effect of go-signal probability on the P3 amplitude compared to N2 amplitude. These results may indicate that the P3 reflects inhibition rather than conflict monitoring. Additionally, they estimated the main source of P3 as the inferior frontal cortex (IFC), which is considered to play an important role in response inhibition, as evidenced by a vast amount of previous literature (e.g., Meffert et al., 2016; Cunillera et al., 2016; Wessel and Aron, 2017). Thus, although disagreement remains over the interpretation of the N2 and P3, they seem to reflect response inhibition and/or conflict.

In relation to RTs and ERP components examined in the Go/No-go task, considerable works reported that they can be influenced by probability of target signals. Generally, RT slows down as the probability of target signals decreases (Bruin and Wijers, 2002; Nieuwenhuis et al., 2003; Hsieh et al., 2016). Also, it has been reported that N2 (Nieuwenhuis et al., 2003; Donkers and Van Boxtel, 2004) as well as P3 (Hsieh et al., 2016) amplitudes can be larger with lower target signal probability. Given these findings, the effect of color on the proactive response inhibition may be affected by the target signal probability. That is, the stronger influence of the color can be predicted with lower probability of target signals.

Accordingly, the purpose of this study was to investigate whether incongruency between prior knowledge of color (traffic lights) and the meaning of presented color would be a cognitive load in the Go/No-go task. To this end, we set up a Blue Go/Red No-go task (i.e., a blue light means to respond while a red light means to refrain) and a Red Go/Blue No-go task with three different target signal probabilities (Go probabilities). In previous literature, reaction time for naming a color is known to be slower when there is a conflict between color name and the color of ink (Stroop effect). Moreover, N2 and P3 amplitudes can be larger with incongruent than congruent stimulus (Pan et al., 2016; Wang et al., 2021). Consequently, we hypothesized that: (1) RTs would be faster when responding to Blue Go than Red Go signal; (2) N2 and P3 amplitudes would be larger in Red Go/Blue No-go than Blue Go/Red No-go task; (3) RTs and N2 and P3 amplitudes would be influenced by the incongruency between prior knowledge of color and the meaning of presented color more strongly in lower Go probability condition.

## Materials and Methods

### Participants

Thirteen healthy participants (4 female, mean age = 28.2 years, SD = 8.5 years) took part in this study, following a previous study (Pomerleau et al., 2014). All participants were strongly right-handed as evaluated by the Edinburg Handedness Inventory scores of 0.9–1.0 (Oldfield, 1971), and had normal or corrected-to-normal vision. Written informed consent was obtained from all participants before beginning the experiment, which was conducted to principles of the Declaration of Helsinki. The study was approved by the Ethics Committee for Clinical Research of Hiroshima University (No. C-242).

### Design and Procedure

**FIGURE 1:**
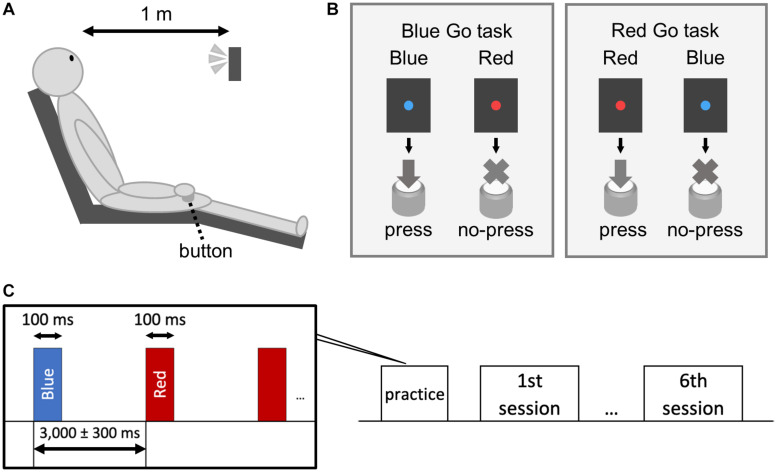
Schematic illustration of the experiment. The subject sat on a chair and performed a Go/No-go task with the right hand **(A)**. We set up a Blue Go/Red No-go task and a Red Go/Blue No-go task **(B)**. The target/non-target signal was presented for 100 ms with an interstimulus interval of 3,000 ± 300 ms **(C)**.

A custom-made light-emitting diode (LED) device (4 Assist, Tokyo, Japan) was used to present blue and red lights (Watanabe et al., 2021). Although the illuminances of blue and red lights were different (7.71 lx for blue light and 4.93 lx for red light), we confirmed that simple RTs to these lights were statistically similar prior to the experiment. The participants faced the LED device set 1 m in front of them at the height of eye (Figure 1A) and performed a Go/No-go task. Blue and red lights were randomly presented for 100 ms at a random interval of 3,000 ± 300 ms (Figures 1B,C) and served as both target (Go) and non-target (No-go) signals. The participants were instructed to press a button held in the right hand as fast as possible when a target (Go) signal appeared and to withhold the response when a non-target (No-go) signal appeared.

The experiment had a 2 × 3 design with the following factors: Color (Blue Go/Red No-go and Red Go/Blue No-go) and Go probability (30, 50, and 70%), resulting in six different conditions. The participants were told which color to respond (Blue or Red) just before each condition session but didn’t know the Go probability. Prior to the sessions, they practiced 30 trials. The condition order was randomized among the participants. Each condition consisted of 100 trials, and sufficient breaks were provided between the condition sessions. RT was calculated as the time from the appearance of Go signal to the pressing of the button. Similar to previous studies (Watanabe et al., 2015; Watanabe et al., 2016a; Rey-Mermet et al., 2019), trials exceeding 3SD from the mean RT of the condition were excluded from statistical analysis.

### EEG Recording and Analysis

Electroencephalogram (EEG) was recorded using three Ag/AgCl active electrodes at Fz, Cz, and Oz according to the International 10–20 system. Eye blinks and movements were monitored via electrooculogram (EOG) using bipolar electrodes attached to the outer side of the right canthus and below the left eye (Watanabe et al., 2016b). All channels were referenced to the linked earlobes. The ground electrode was attached to the left forearm using the disposable gel electrode (GE Health Care Japan, Tokyo, Japan). The EEG and EOG were amplified (BA1008; Nihon Sankeku, Osaka, Japan), filtered between 0.1 and 100 Hz, and digitized at sampling rate of 1 kHz. Impedance was kept below 10 kΩ. The custom-made LED device was programmed to send a pulse trigger for synchronization with EEG (4 Assist, Tokyo, Japan). Continuous EEG data were segmented into 1,000 ms epochs starting 100 ms prior to the stimulus onset. Epochs exceeding ±100 μV were automatically discarded. Furthermore, we visually inspected and discarded epochs still contaminated by artifacts (Ozubko et al., 2021). The average number of discarded epochs was 14 ± 12, and the average number of retained epochs was 36 ± 17. Following previous studies (Schoenberg et al., 2014; Nguyen et al., 2016), a threshold to exclude subjects from the analysis was set as five. The artifact-free epochs were then averaged separately for Go and No-go trials in each condition in order to obtain ERP components. Subsequently, we identified the following peaks: P1 from 100 to 170 ms, N2 from 130 to 300 ms, and P3 from 250 to 500 ms for the front-central site (Fz and Cz), and C1 from 50 to 110 ms, P2 from 180 to 250 ms, N2 from 200 to 300 ms, and P3 from 250 to 500 ms for the occipital site (Oz). Using these peaks, we finally calculated the C1, N2, and P3 amplitudes and latencies. The C1 amplitude was defined as the difference between C1 peak and a baseline (–100 to 0 ms), the N2 amplitude was defined as the difference between the P1 (Fz and Cz) or P2 (Oz) and N2 peaks, and the P3 amplitude was defined as the difference between the N2 and P3 peaks (peak-to-peak measurements). As the minimum number of artifact-free epochs accepted into condition averages in this study was seven (range 7–70), all conditions from all participants were included in the statistical analysis.

### Statistical Analysis

SPSS Statistics software version 21 (SPSS; IBM Corp., Armonk, NY, United States) was used for statistical analysis. A two-way repeated-measures analysis of variance (ANOVA) was used to determine the effect of Color (Blue Go and Red Go) and Go probability (30, 50, and 70%) on the mean RT and ERP amplitude and latency. Furthermore, Pearson’s correlation coefficients were calculated between the RT and ERP amplitude or latency. *Post hoc* test was conducted with Bonferroni adjustment. Significant level was set at *p* < 0.05. The effect size for each ANOVA was calculated using partial eta squared (partial η^2^).

## Results

### Behavioral Results

The average number of trials in which the participants did not respond to Go signals (Go omission errors) and that in which the participants responded to No-go signals (No-go commission errors) are presented in Table 1. These trials were not included in RT or ERP analysis.

**TABLE 1:** Go omission and No-go commission errors (mean ± SD).

	Blue Go 30%/Red No-go 70%	Blue Go 50%/Red No-go 50%	Blue Go 70%/Red No-go 30%	Red Go 30%/Blue No-go 70%	Red Go 50%/Blue No-go 50%	Red Go 70%/Blue No-go 30%
Go omission errors	0.77 ± 1.77	1.08 ± 1.75	1.08 ± 1.50	0.46 ± 0.97	0.54 ± 1.20	1.23 ± 3.85
No-go commission errors	0.31 ± 0.63	0.31 ± 0.63	0.46 ± 0.52	0.23 ± 0.44	0.69 ± 0.75	0.77 ± 1.24

**FIGURE 2:**
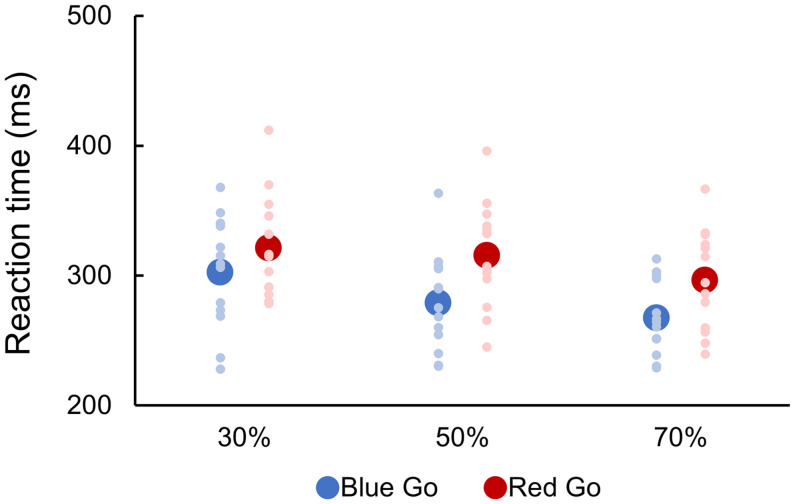
Reaction time to go signal. There were six conditions created by crossing two target colors (Blue Go/Red No-go and Red Go/Blue No-go) and three target probabilities (30, 50, and 70%). Individual data (light color) and their mean (dark color) were presented for each condition.

Mean RTs are depicted in Figure 2. A two-way repeated-measures ANOVA indicated significant main effects of Color [*F*(1, 12) = 22.933, *p* < 0.001, partial η^2^ = 0.656] and Go probability [*F*(2, 24) = 25.373, *p* < 0.001, partial η^2^ = 0.679], but there was no significant interaction between them [*F*(2, 24) = 0.911, *p* = 0.384, partial η^2^ = 0.071]. *Post hoc* analyses revealed that the higher Go probability, the faster RT (*p* < 0.05).

### ERP Results

**FIGURE 3:**
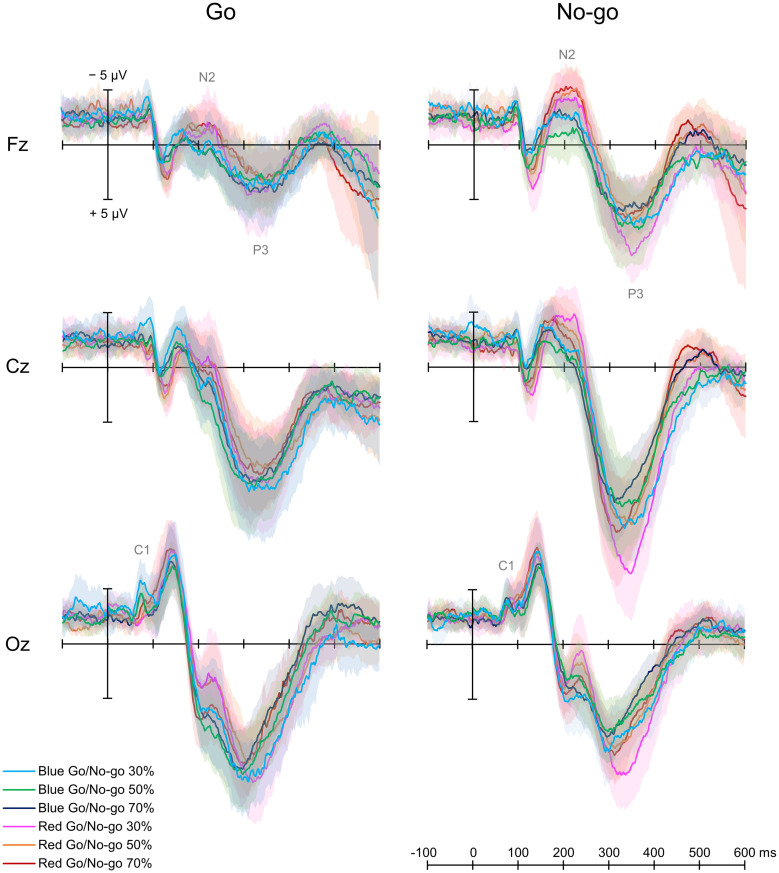
Grand average event-related potential waveforms. There were six conditions created by crossing two target colors (Blue Go/Red No-go and Red Go/Blue No-go) and three target probabilities (30, 50, and 70%).

Figure 3 shows grand average ERP waveforms for six conditions.

#### Early Component

A two-way repeated-measures ANOVA showed a main effect of Color [*F*(1, 12) = 5.747, *p* = 0.020, partial η^2^ = 0.485] and an interaction between Color and Go probability [*F*(2, 24) = 4.317, *p* = 0.018, partial η^2^ = 0.329] on C1 amplitude for Go ERPs. A *post hoc* analysis revealed that C1 amplitude was larger in Blue Go than Red Go trial in 30% Go probability. Regarding the C1 latency, there was no significant main effect or interaction for Go ERPs.

#### N2 and P3 Amplitudes

**FIGURE 4:**
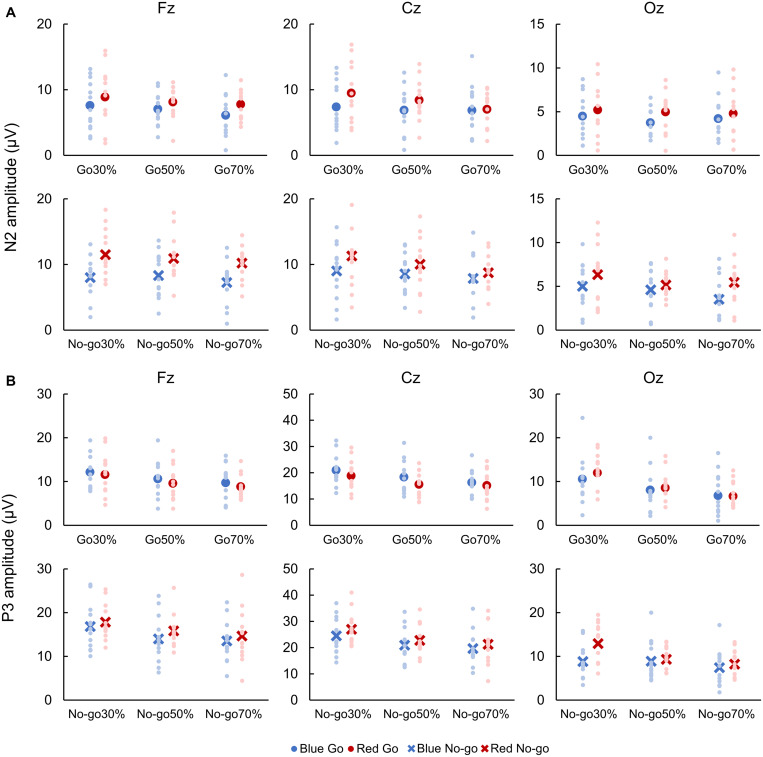
Amplitudes of N2 **(A)** and P3 **(B)**. Blue color indicates responses to a blue light (Blue Go and Blue No-go), and red color indicates responses to a red light (Red Go and Red No-go). Individual data (light color) and their mean (dark color) were presented for each condition.

For both Go and No-go ERPs, a two-way repeated-measures ANOVA showed a main effect of Color on N2 amplitude, which was larger when responding to Red Go than Blue Go and when withholding a response to Red No-go than Blue No-go at Fz [Go: *F*(1, 12) = 5.961, *p* = 0.018, partial η^2^ = 0.337; No-go: *F*(1,12) = 33.525, *p* < 0.001, partial η^2^ = 0.837], Cz [Go: *F*(1, 12) = 6.120, *p* = 0.016, partial η^2^ = 0.303; No-go: *F*(1, 12) = 6.620, *p* = 0.013, partial η^2^ = 0.431], and Oz [Go: *F*(1,12) = 7.444, *p* = 0.009, partial η^2^ = 0.090; No-go: *F*(1, 12) = 7.770, *p* = 0.007, partial η^2^ = 0.218]. There was also a main effect of Go probability on N2 amplitude for No-go ERPs at Cz [*F*(2, 24) = 3.185, *p* = 0.048, partial η^2^ = 0.199], and a *post hoc* analysis revealed that the N2 amplitude was larger in 30% than 70% No-go probability (*p* = 0.043, Figure 4A).

With respect to P3 amplitude, for both Go and No-go ERPs, a two-way repeated-measures ANOVA showed a main effect of Color at Cz [Go: *F*(1, 12) = 7.739, *p* = 0.007, partial η^2^ = 0.250; No-go: *F*(1, 12) = 4.362, *p* = 0.041, partial η^2^ = 0.124] and Oz [Go: *F*(1, 12) = 6.069, *p* = 0.018, partial η^2^ = 0.247; No-go: *F*(1, 12) = 45.808, *p* < 0.001, partial η^2^ = 0.774]. Also, there was a main effect of Go probability on P3 amplitude for Go ERPs at Fz [*F*(2, 24) = 5.137, *p* = 0.009, partial η^2^ = 0.316] and Cz [*F*(2, 24) = 11.559, *p* < 0.001, partial η^2^ = 0.597], and for No-go ERPs at Fz [*F*(2, 24) = 6.863, *p* = 0.002, partial η^2^ = 0.462], Cz [*F*(2, 24) = 11.388, *p* < 0.001, partial η^2^ = 0.609], and Oz [*F*(2, 24) = 15.296, *p* < 0.001, partial η^2^ = 0.693]. We further found an interaction between Color and Go probability on P3 amplitude for No-go ERPs at Oz [*F*(2, 24) = 5.536, *p* = 0.006, partial η^2^ = 0.283]. *Post hoc* analyses demonstrated that the P3 amplitude for Go ERPs was larger in 30% than 70% Go probability at both Fz and Cz (*p* < 0.01), and that the P3 amplitude for No-go ERPs was larger in 30% than 50 and 70% No-go probability at Fz and Cz (*p* < 0.05). For No-go ERPs at Oz, *post hoc* analyses revealed that the P3 amplitude was larger in Red No-go than Blue No-go trial in 30% No-go probability (*p* = 0.007), and that it was larger in 30% than 50% (*p* = 0.004) and 70% (*p* < 0.001) No-go probability in Red No-go trial (Figure 4B).

#### N2 and P3 Latencies

**FIGURE 5:**
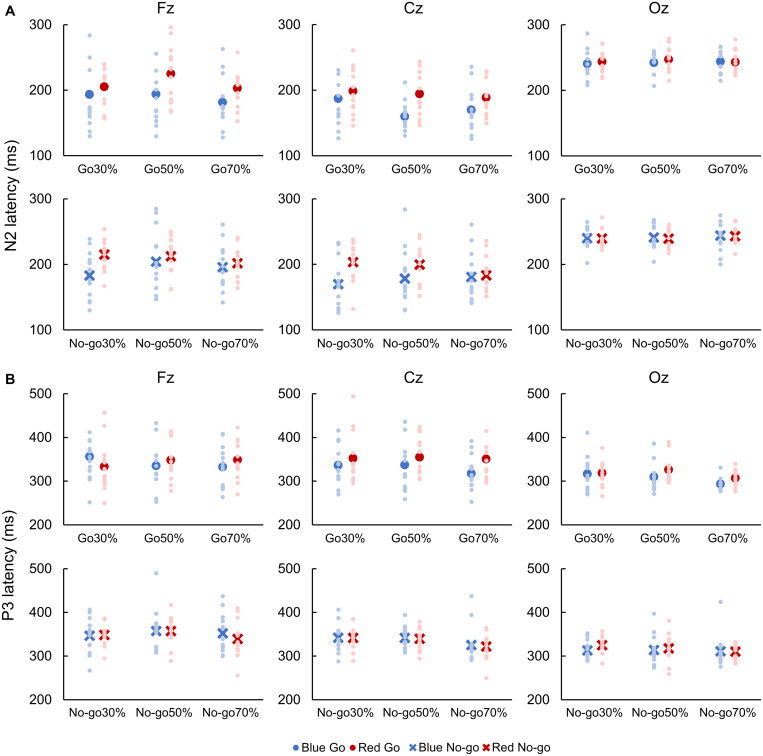
Latencies of N2 **(A)** and P3 **(B)**. Blue color indicates responses to a blue light (Blue Go and Blue No-go), and red color indicates responses to a red light (Red Go and Red No-go). Individual data (light color) and their mean (dark color) were presented for each condition.

For both Go and No-go ERPs, a two-way repeated-measures ANOVA showed a main effect of Color on N2 latency, which was faster when responding to Blue Go than Red Go and when withholding a response to Blue No-go than Red No-go at Fz [Go: *F*(1, 12) = 7.041, *p* = 0.010, partial η^2^ = 0.426; No-go: *F*(1, 12) = 7.533, *p* = 0.008, partial η^2^ = 0.236] and Cz [Go: *F*(1, 12) = 9.504, *p* = 0.003, partial η^2^ = 0.451; No-go: *F*(1, 12) = 8.678, *p* = 0.005, partial η^2^ = 0.306; Figure 5A].

A two-way repeated-measures ANOVA revealed a main effect of Color on P3 latency for Go ERPs at Cz [*F*(1, 12) = 6.376, *p* = 0.014, partial η^2^ = 0.210]. Also, there was a main effect of Go probability on P3 latency for Go ERPs at Oz [*F*(2, 24) = 6.110, *p* = 0.004, partial η^2^ = 0.363] and for No-go ERPs at Cz [*F*(2, 24) = 5.305, *p* = 0.008, partial η^2^ = 0.318]. *Post hoc* analyses revealed that the P3 latency for Go ERPs was faster in 70% than 30 and 50% Go probability at Oz. Conversely, P3 latency for No-go ERPs was faster in 70% than 30 and 50% No-go probability at Cz (*p* < 0.05; Figure 5B).

### Correlations Between RT and ERP Amplitude/Latency

Table 2 shows correlations between the RT and N2 or P3 amplitude. Significant positive correlations were obtained between the RT and N2 amplitude at Fz for Go ERPs, and at Oz for No-go ERPs. There was a significant negative correlation between the RT and P3 amplitude at Cz for Go ERPs.

**TABLE 2:** Correlation between RT and ERP amplitude.

	Go N2	No-go N2	Go P3	No-go P3
Fz	0.369	0.196	–0.134	–0.125
Cz	0.315	0.220	–0.338	–0.173
Oz	0.320	0.403	–0.054	0.228

Table 3 shows correlations between the RT and N2 or P3 latency. We found significant positive correlations between the RT and P3 latency at Cz and Oz for Go ERPs.

**TABLE 3:** Correlation between RT and ERP latency.

	Go N2	No-go N2	Go P3	No-go P3
Fz	0.289	–0.034	0.157	0.033
Cz	0.292	0.095	0.350	0.232
Oz	0.159	0.274	0.541	0.314

## Discussion

The present study aimed to elucidate the effect of prior knowledge of color on RTs and ERPs during a Go/No-go task. For that purpose, we set up Blue Go/Red No-go and Red Go/Blue No-go tasks with three different Go probabilities (30, 50, and 70%). Overall, we found the slower RTs in Red Go than Blue Go trial, and also with lower Go probability. Furthermore, in general, amplitudes of N2 and P3 components of ERPs were larger in Red Go/Red No-go than Blue Go/Blue No-go trial and were larger with lower Go/No-go probability.

We found that the RT was faster in Blue Go than Red Go trial, and this result may be related to the Stroop effect (Stroop, 1935), in which the naming of written color words is impeded by the occurrence of different ink color. In a typical Stoop task with the conflict between a word name and its ink color, for instance, participants can respond faster to a letter “Red” printed in red than the one printed in blue. In addition, the Stroop-like effect has been reported using a conflict between color and shape of pedestrian traffic sign (e.g., walking sign in red color) (Peschke et al., 2013). On the other hand, in a simple RT task using written color words printed in congruent or incongruent color as target stimuli (e.g., a letter “Blue” printed in blue ink vs. a letter “Green” printed in yellow ink), RTs to congruent and incongruent words were found to be similar (Gonzalez-Rosa et al., 2013).

Moreover, in a previous study using a color-object verification task, surface color of an object was found to activate relevant semantic knowledge about the object, which impacted RTs (Bramão et al., 2012). Specifically, when asked to judge whether object’s color was typical or atypical, RTs to objects with typical color were faster than ones with atypical color. Meanwhile, when asked to judge whether the surface color of an object (typical or atypical) was matched or unmatched with the color name presented beforehand while ignoring the prototypical color of the object, there was no significant difference in the RT between typical and atypical colors. Collectively, these findings indicate that RTs can be influenced selectively by conflict between prior knowledge about objects’ color and the presented color of the objects.

In the present study, therefore, the Stroop-like interference caused by information conflict between prior knowledge about traffic light signals and the meaning of presented color likely delayed the response to a signal in Red Go trials. Considering this finding in the context of real-world car driving, drivers need to recognize that responding to a red signal can be slower than responding to a green/blue signal and thus must not start pressing the brake pedal after the traffic signal turns red; they should start pressing the brake pedal when the traffic light turns yellow (to prevent a delayed response). Extending this study further by using driving simulators would provide more detailed information in the future.

With respect to Go probability, the RTs became slower as Go probability decreased, which is consistent with previous studies (Braver et al., 2001; Bruin and Wijers, 2002; Nieuwenhuis et al., 2003; Hsieh et al., 2016; Meffert et al., 2016). This result indicates that lower target-signal probability conditions are more difficult as there is a bias toward the high probability No-go response (refraining from responding) (Braver et al., 2001; Meffert et al., 2016). Contrarily, response preparation is enhanced in higher target-signal probability conditions (Low and Miller, 1999), allowing a faster response. Taken as a whole, our findings of RTs being slower in Red Go than Blue Go trials and also with lower Go probability agree with well-known notion that RTs can be influenced by conflicts and/or cognitive load (Peschke et al., 2013; Nieuwenhuis et al., 2003). This study adds to the current literature by demonstrating that prior knowledge of color can be a conflict when proactive response inhibitory function is required during a Go/No-go task. We suppose that experiment design used in this study can be a simple and effective way to manipulate cognitive load of Go/No-go task.

In regard to the ERP components, the C1 amplitude was larger in Blue Go than Red Go trial in 30% Go probability. In a previous study by Eason et al. (1967), amplitude of occipital ERP component around 50–100 ms was found to be larger with higher luminous intensity and when responding to a red than blue light in a simple RT task. Thus, the difference in C1 amplitude found in this study may be due to the difference in the luminous intensity of LED between blue and red lights. Meanwhile, simple RTs were revealed to be unaffected by light color (Eason et al., 1967), and we preliminarily found that they were unaffected by the luminous intensity of LEDs used in this study. Therefore, the C1 amplitude difference observed here likely did not contribute to the changes in RTs (in Go/No-go task). Indeed, the C1 originates in the primary visual cortex (Clark and Hillyard, 1996), and we found insignificant difference in the C1 latency. It appears that the visual stimulus was similarly processed at least to the primary visual cortex. Accordingly, the difference in task performance between Blue Go and Red Go trials can be attributed to changes in the higher-order processing shown in N2 and P3 amplitudes, as discussed below.

First, we demonstrated that the N2 amplitude was larger when responding to red than blue light in Go trials. This result can be attributed to a conflict between prior knowledge of color and the meaning of presented color, as N2 amplitude has been shown to be larger in incongruent than congruent trial in the Stroop task (Boenke et al., 2009; Pan et al., 2016). In a recent review by Heidlmayr et al. (2020), the cortical origin of N2 was considered to be the ACC, IFC, and/or prefrontal cortex. It has been reported that the ACC detects the presence of conflict (Botvinick et al., 2001), subsequently engaging the dorsolateral prefrontal cortex to impose cognitive control to resolve the conflict (Parris et al., 2019), whereas the IFC is responsible for processing of both response and semantic conflicts (Parris et al., 2019). Therefore, our finding of the larger N2 amplitude in Red Go than Blue Go trial may be due to the stronger activation of these brain regions. In addition to the amplitude, the N2 latency for Go ERPs was found to be faster in Blue Go than Red Go trial, which seems to be in line with a view that N2 latency reflects processing time of response selection (Gajewski et al., 2008).

In contrast to our expectation, N2 amplitude for Red No-go trials was larger than that for Blue No-go trials. Although hard to interpret, this result may be attributed to greater experience of inhibition with red light signals, as N2 amplitude for No-go trials is suggested to be related to attention and modulated by physical training (Yamashiro et al., 2015). For example, N2 amplitude for No-go trials was larger in fencers (Di Russo et al., 2006) and baseball players (Yamashiro et al., 2015) compared with controls. Even though the participants of the present study have not undergone any special training like top athletes, they have a great amount of experience to choose right actions according to the color of traffic lights. Thus, this experience might have enhanced the N2 amplitude for Red No-go trials. In addition, several studies have reported that N2 amplitude for No-go trials negatively correlates with RT, meaning that the shorter RTs are associated with the larger N2 amplitude for No-go trials (Yamashiro et al., 2015). The stronger inhibitory function reflected by the larger N2 amplitude is thought to result in the shorter RT (Band et al., 2003; Yamashiro et al., 2015). In the present study, the RT was faster and the N2 amplitude for No-go trials was larger in Blue Go/Red No-go than Red Go/Blue No-go task, although no significant correlation was found between the RT and N2 amplitude for No-go trials at front-central sites. Thus, we suppose that a stronger inhibitory function was recruited in Red No-go than Blue No-go trial, and this inhibitory function may have partially influenced the RTs.

Next, we would like to discuss about P3 amplitude, which is associated with a number of different cognitive mechanisms. Similar to previous studies (e.g., Hsieh et al., 2016), the P3 amplitude was influenced by Go probability in both Go and No-go trials. Also, it was larger in Red No-go than Blue No-go trial at Cz and Oz in some cases. Given that P3 reflects the amount of allocated attention (Luck and Kappenman, 2011), the larger P3 amplitude in Red No-go trial may indicate that it was easier to pay attention to Red No-go than Blue No-go signal because of participants’ familiarity with traffic light signals. With regard to RTs, a shorter RT has been reported to be associated with a larger P3 amplitude for No-go trials (Yamashiro et al., 2015), similar to the N2, and Nakata et al. (2012) suggested that a faster response to Go signal can occur as a result of a larger No-go related neural activity. Therefore, considering that P3 can reflect response inhibition (Enriquez-Geppert et al., 2010), the larger P3 amplitude for No-go trial in Blue Go/Red No-go than Red Go/Blue No-go task may have led to the faster response to Go signal in this study. On the other hand, although the neurophysiological mechanisms underlying P3 have been explored in a number of studies, no clear consensus has been reached on this matter, making the interpretation of P3 complicated and difficult (for reviews, Gupta et al., 2019; see also, Luck, 2014). In a review by Polich (2007), they showed that P3 is made up of several subcomponents including the frontal maximal P3a and temporal-parietal maximal P3b. The P3a may originate from the dorsolateral prefrontal cortex, IFC, and cingulate cortex, and can be influenced by stimulus probability, while the P3b may originate from the ventrolateral prefrontal cortex, superior temporal sulcus, and intraparietal sulcus and index a response to a target signal (Halgren et al., 1998). Yet, we could not detect these two subcomponents in this study, warranting future studies to better understand the functional role of P3 and its subcomponents in response inhibition as well as color conflict.

Finally, we would like to consider how our findings can be translated to clinical application. One possible way is an assessment of driving function of individuals with potential mild cognitive impairment (MCI), as their cognitive processing speed is highly associated with driving functions (Wadley et al., 2020; Toepper et al., 2021). During driving license renewal for the elderly, questionnaires and driving simulations are typically used to assess their driving function, and no neurophysiological assessments are performed. Meanwhile, recent studies have reported that abnormal ERPs can be a biomarker for detecting cognitive decline, particularly of verbal memory, in elderly individuals with preclinical Alzheimer’s disease (Olichney et al., 2013) and MCI (Xia et al., 2020). In addition, ERPs associated with a Go/No-go task were found to be compromised in individuals with MCI (López Zunini et al., 2016). Therefore, the task and ERP measurements used in the present study could be a simple and efficient method to manipulate the cognitive load to detect a subtle cognitive decline that may cause bewilderment and delay in responses during driving. Further studies and technological advances are required to promote this field of research.

There are limitations that should be acknowledged in this study. First, the sample size was small; thus, future studies with larger sample sizes may be warranted to test our findings. Second, the minimum number of trials used to create the averaged waveform was seven. Although the threshold was set according to previous ERP studies (Schoenberg et al., 2014; Nguyen et al., 2016), it is recommended to include approximately 20 trials in another study (Cohen and Polich, 1997). Also, a recent study suggests that the number of trials for averaging should be increased especially when the sample size is small (Boudewyn et al., 2018). Therefore, caution may be needed when interpreting our ERP results. Due to the issues of sample size and minimum trials used for conditions, results of this study should be taken as preliminary and used to motivate future studies until they are able to be independently reproduced or replicated in a much larger sample size of participants and using a larger sample of valid trials for inclusion in ERP analyses.

In summary, we found that RT was slower and N2 amplitude was larger when making a response to red than blue light in a Go/No-go task, and these findings was interpreted as a Stroop-like interference, that is, a conflict between prior knowledge about traffic light signals and the meaning of presented signal. In addition, N2 and P3 amplitudes were larger in Red No-go than Blue No-go trial, which might have been induced by years of experience in stopping an action in response to a red signal and/or attention. This study provides the better understanding of the effect of prior knowledge of color on behavioral responses and its underlying neural mechanisms.

## Data Availability Statement

The original contributions presented in the study are included in the article/supplementary materials, further inquiries can be directed to the corresponding author/s.

## Ethics Statement

The studies involving human participants were reviewed and approved by the Ethics Committee for Clinical Research of Hiroshima University. The patients/participants provided their written informed consent to participate in this study.

## Author Contributions

NK, TW, and HK designed the study, edited, and revised the manuscript. NK and XC performed the experiment. NK analyzed the data and wrote the original draft of the manuscript. XC, TM, KY, and TK assisted the data analysis and the preparation of the manuscript. TW and HK supervised the study. All authors approved the final version of the manuscript.

## Conflict of Interest

The authors declare that the research was conducted in the absence of any commercial or financial relationships that could be construed as a potential conflict of interest.
